# Transcranial brain atlas‐based optimization for functional near‐infrared spectroscopy optode arrangement: Theory, algorithm, and application

**DOI:** 10.1002/hbm.25318

**Published:** 2020-12-17

**Authors:** Yang Zhao, Xiang Xiao, Yi‐Han Jiang, Pei‐Pei Sun, Zong Zhang, Yi‐Long Gong, Zheng Li, Chao‐Zhe Zhu

**Affiliations:** ^1^ State Key Laboratory of Cognitive Neuroscience and Learning Beijing Normal University Beijing China; ^2^ Neuroimaging Research Branch, National Institute on Drug Abuse National Institutes of Health Baltimore Maryland USA; ^3^ Center for Cognition and Neuroergonomics, State Key Laboratory of Cognitive Neuroscience and Learning Beijing Normal University at Zhuhai Zhuhai China; ^4^ IDG/McGovern Institute for Brain Research Beijing Normal University Beijing China; ^5^ Center for Collaboration and Innovation in Brain and Learning Sciences Beijing Normal University Beijing China

**Keywords:** fNIRS, functional near‐infrared spectroscopy, navigation, optode arrangement, optode montage design, optode placement, topography, transcranial brain atlas

## Abstract

The quality of optode arrangement is crucial for group imaging studies when using functional near‐infrared spectroscopy (fNIRS). Previous studies have demonstrated the promising effectiveness of using transcranial brain atlases (TBAs), in a manual and intuition‐based way, to guide optode arrangement when individual structural MRI data are unavailable. However, the theoretical basis of using TBA to optimize optode arrangement remains unclear, which leads to manual and subjective application. In this study, we first describe the theoretical basis of TBA‐based optimization of optode arrangement using a mathematical framework. Second, based on the theoretical basis, an algorithm is proposed for automatically arranging optodes on a virtual scalp. The resultant montage is placed onto the head of each participant guided by a low‐cost and portable navigation system. We compared our method with the widely used 10/20‐system‐assisted optode arrangement procedure, using finger‐tapping and working memory tasks as examples of both low‐ and high‐level cognitive systems. Performance, including optode montage designs, locations on each participant's scalp, brain activation, as well as ground truth indices derived from individual MRI data were evaluated. The results give convergent support for our method's ability to provide more accurate, consistent and efficient optode arrangements for fNIRS group imaging than the 10/20 method.

## Graphical abstract

The theoretical basis of transcranial brain atlas‐based optimization of optode arrangement is illustrated using a mathematical framework. Based on the theoretical basis, optodes are automatically arranged on a virtual scalp and the resultant optode montage is placed for each participant guided by a navigation system for each participant. The results of validation experiments indicate our method outperforms the traditional 10/20‐based method in both optode arrangement quality and task activation results.

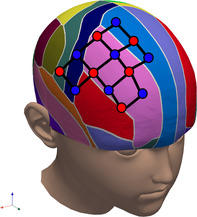

## 1 INTRODUCTION

Functional near‐infrared spectroscopy (fNIRS) is a non‐invasive imaging technique which has been widely applied in the study of human brain functions (Boas, Elwell, Ferrari, & Taga, 2014). Being portable, few in restrictions and in‐sensitive to body movements, fNIRS offers high ecological validity and is suitable for conducting experiments in natural environments. Due to these advantages, fNIRS can provide new insights for social neuroscience via hyperscanning studies and can be easily applied to special populations such as infants, patients ptand the elderly (Chen et al., 2017; Ferrari et al., 2012; Hari et al., 2015; Monden et al., 2012). FNIRS also provides a more comprehensive measurement of hemoglobin concentration changes, including oxygenated hemoglobin (HbO_2_), deoxygenated hemoglobin, and cerebral blood volume together with a higher temporal sampling rate than functional magnetic resonance imaging (fMRI) (Scholkmann et al., 2014). Therefore, it has also contributed to a deeper understanding of neurovascular coupling and the origin of the BOLD signal via fNIRS‐fMRI studies (Scarapicchia, Brown, Mayo, & Gawryluk, 2017; Steinbrink et al., 2006).

FNIRS imaging can be applied in either topographic or tomographic mode. Tomographic fNIRS, also called diffuse optical tomography (DOT), can provide 3D images with higher spatial resolution than conventional 2D topographic fNIRS (Bluestone, Abdoulaev, Schmitz, Barbour, & Hielscher, 2001; Boas & Dale, 2005; Zeff, White, Dehghani, Schlaggar, & Culver, 2007). However, it requires fNIRS devices which supporting numbers of flexibly arranged optodes and application and analysis are often arduous (Pinti et al., 2020). Therefore, topographic fNIRS, or diffuse optical imaging (DOI), is more accessible and has been applied extensively in the fNIRS literature. In topographic fNIRS experiments, the optodes are often arranged with a certain spatial pattern and source‐detector distance to image a spatially extensive brain area. To derive group activation results, DOI studies often average effects recorded on a set of channels across a group of participants, which is often called channel‐wise or sensor space analysis. Therefore, optode montages should be carefully placed to ensure the consistency of brain location measured by each channel across participants. At the same time, because the number of optodes is often limited, optodes should be arranged at scalp locations which can cover the regions of interest (ROIs) specified in the study.

To image underlying brain areas by placing optodes on the scalp, it is mandatory to refer to the scalp‐brain correspondence for fNIRS. Theoretically, since there is interindividual variation in scalp‐brain correspondence, the optimal optode arrangement for each participant can only be decided based on the individual‐specific scalp‐brain correspondence (Machado et al., 2018). However, to image a group of participants, obtaining individual scalp‐brain correspondences using MRI scans is time consuming and cost‐ineffective. More importantly, it makes fNIRS dependent on an MRI scanner, which reduces the accessibility of fNIRS (Singh, Okamoto, Dan, Jurcak, & Dan, 2005). Therefore, fNIRS studies often arrange optodes for a group of participants based on substitutive scalp‐brain correspondences, acquired either from head templates or sMRI images of a population.

The most prevalent and easy‐to‐use approach for fNIRS optode arrangement in the literature is according to the probabilistic scalp‐brain correspondence of the international 10/20 system, established by both Homan and Okamoto (Homan, Herman, & Purdy, 1987; Okamoto et al., 2004). The 10/20 landmarks can be manually measured on participants' scalps, so researchers can select ROI related to 10/20 landmarks and thereby place the optode montage for a group of participants in an fNIRS study. However, fNIRS imaging systems often offer multichannel measurement probe sets that can be arranged at any scalp location with arbitrary orientation. Therefore, it is challenging to optimize optodes to best record from ROI using only the sparse scalp‐brain correspondence of 10/20 landmarks. Later, studies proposed methods for optimizing optode arrangement. Using a 3D‐printed ICBM152 physical head model and optical navigation system, a probe set can be manually navigated to cover ROI on a head template according to the scalp‐brain correspondence provided by the navigation system, then transferred to the heads of participants using the 10/20 system (Cutini, Scatturin, & Zorzi, 2011). AtlasViewer also enables registration of the locations of arranged optodes to a head template and evaluation of the measurement sensitivity for a given ROI (Aasted et al., 2015). Therefore, researchers can iteratively modify the probe arrangement to increase sensitivity using the toolbox (Wijeakumar, Spencer, Bohache, Boas, & Magnotta, 2015). Morais et al. offer the FOLD toolbox for automatically deciding optode arrangement (Morais, Balardin, & Sato, 2018). Although these approaches enable optimizing the optode arrangement for a given ROI, they remain difficult to use in practice. The former two methods involve additional instruments such as a 3D‐digitizer system and physical head model, as well as manual operation for arranging or modifying the probe set, which is time consuming and may lead to optode arrangement results sensitive to human error. The FOLD toolbox only supports placing optodes at sparse head locations, that is, 10/20, 10/10, and 10/5 landmarks; therefore, it can only be used for specific fNIRS systems. Moreover, these methods optimize the optode arrangement based on the scalp‐brain correspondence of a single head template, which can only produce optimal arrangement results for that head template, instead of the group of participants to be imaged.

Transcranial brain atlases (TBAs) are digital atlases which provide high‐resolution probabilistic scalp‐brain correspondences based on large samples of individuals (Xiao et al., 2018). To apply the constructed TBA for optode arrangement, previous studies used manual arrangement of optode montages on a head model assisted by a custom‐made scalp navigation system. Promising results based on TBA‐assisted probe arrangement have been demonstrated (Xiao et al., 2018). However, due to a lack of theoretical basis, the manual optimization procedure is often intuition‐based, subjective, and time‐consuming. It also makes the head model, as well as a 3D‐digitizer, indispensable for applying the TBA‐based optode arrangements. In this study, we first describe the theoretical basis of TBA‐based optimization of optode arrangement using a mathematical framework. Specifically, we propose two indices for topographic fNIRS, group imaging variability (GIV) and group imaging accuracy (GIA), to evaluate an optode arrangement's quality based on individual scalp‐brain correspondence. Using the probabilistic scalp‐brain correspondences provided by a TBA, we further show these two indices can be optimized. Second, we propose an algorithm for automatic arrangement of optode montages, and a protocol for placing optode montages via a navigation system. To validate the methods, motor and working memory functions are selected as targets for recording under the proposed method for a group of participants. We compare our method with the widely used 10/20‐based optode arrangement method.

## 2 THEORY AND METHODS

### 2.1 Evaluation indices for optode arrangement quality

We propose two indices to evaluate the optode arrangement quality based on an individual's scalp‐brain correspondence, which can be computed from the individual's sMRI of participants. In an fNIRS study, researchers often image a group of participants using one optode montage, which includes multiple data acquisition channels. Here, we denote the number of participants and the number of channels as *N*_*p*_ and *N*_*c*_, respectively. For each participant *j*, the scalp location of the *i*th channel, assumed to be the mid‐point between the source and detector, is denoted as *s*_*ij*_. The brain location measured can be obtained by projecting *s*_*ij*_ to the brain surface of the participant, which is denoted as *b*_*ij*_ (Okamoto & Dan, 2005). The consistency of measured brain locations across participants is important for the reliability of activation results obtained in an fNIRS study. Therefore, we propose the GIV for the *i*th channel to evaluate the variability of *b*_*ij*_ in standard brain space (e.g., MNI space) across participants:

(1) $${\mathrm{GIV}}_{i}=\sqrt{\frac{1}{N_{p}-1}\sum_{j=1}^{N_{p}}{\left(x_{j}-\bar{x}\right)}^{2}+{\left(y_{j}-\bar{y}\right)}^{2}+{\left(z_{j}-\bar{z}\right)}^{2}}$$

Here, *b*_*ij*_ = (*x*_*j*_, *y*_*j*_, *z*_*j*_) are MNI coordinates of the measurement locations for the *j*th participant. GIV reflects the average dispersion of the measurement locations. The GIV for an optode montage of many channels is defined as the average across channels:

(2) $$\mathrm{GIV}=\frac{1}{N_{c}}\sum_{i}^{N_{c}}{\mathrm{GIV}}_{i}$$

A lower value of *GIV* means higher consistency in the measurement locations of the optode arrangement across participants.

For the *i*th channel of the *j*th participant, the (anatomical or functional) label of the channel, denoted as *L*_*ij*_, can be obtained from macroanatomical brain atlases (e.g., Brodmann area [BA] or automatic anatomical labeling [AAL]) or through meta‐analysis (Tsuzuki & Dan, 2014). Usually, one or more brain ROIs are studied in an fNIRS experiment. Whether measurement locations are within the ROI is another important criterion for optode arrangement, so we propose an index to evaluate it. Here, we let *L*_*ij*_ = 1 if *L*_*ij*_ ∈ *ROIs*, otherwise *L*_*ij*_ = 0. The GIA for the *i*th channel is then:

(3) $${\mathrm{GIA}}_{i}=\frac{\sum_{j=1}^{N_{p}}L_{\mathrm{ij}}}{N_{p}}$$

It is the percentage of participants whose *i*th channel fall within the ROIs. The GIA of an optode montage is the average across all channels:

(4) $$\mathrm{GIA}=\frac{1}{N_{c}}\sum_{i}^{N_{c}}{\mathrm{GIA}}_{i}$$

A higher value of GIA means there are more participants whose measured locations lie within the ROI and thus imaging accuracy is higher for the optode arrangement.

### 2.2 TBA‐based optimization theory

#### 2.2.1 Optimization of GIV using TBA

Optimization based on GIA and GIV can find theoretically best‐performing optode arrangements. However, it involves individual scalp‐brain correspondences obtained from sMRI images of participants to be imaged, which are not commonly available in fNIRS studies. This hinders the direct usage of GIA and GIV for optode arrangement. An alternative way to optimize GIV and GIA is to use the probabilistic scalp‐brain correspondence given by a TBA.

**FIGURE 1:**
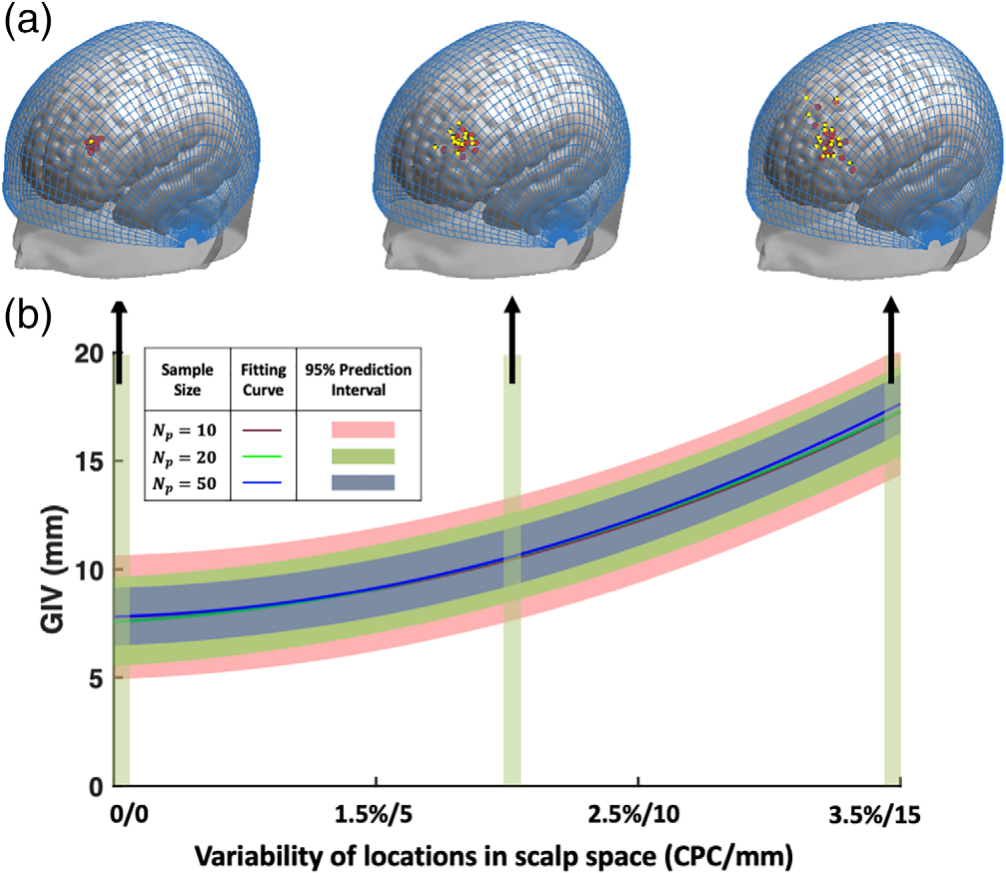
Relationship between location variability in scalp space and brain space. (a) Representative scalp locations (yellow dots) and their corresponding brain projections (red dots) of *N*_*p*_ = 20 participants, which are displayed on the MNI152 head template with overlayed continuous proportional coordinates (CPCs) (blue grid). (b) Correlation between variability in scalp space and brain space. Different colored curves represent different numbers of participants (*N*_*p*_) used in evaluating the relationship

To minimize GIV (defined in brain space) without using participant‐specific sMRI, one can minimize variability of the channel locations in scalp space across participants. Given scalp surface points and fiducial marks, that is, Nasion (Nz), Inion (Iz), left preauricular (AL), and right preauricular (AR), the continuous proportional coordinates (CPC) of points on the scalp surface of participants can be automatically measured based on a previously proposed procedure (Xiao et al., 2018). A CPC grid measured on the MNI 152 scalp template is shown in Figure 1a. CPC can be used to characterize channel locations and quantify the variability of locations in scalp space across participants. Scalp location variability can be defined either based on CPCs or the variability of 3D coordinates (as GIV does) on a typical scalp. The relationship between the variability in scalp space and brain space was experimentally evaluated and results are shown in Figure 1 (details in the Supplementary Material). In general, the GIV in brain space monotonically increases as the variability of locations increases in scalp space. With an increase in the number of participants used during construction of the scalp‐brain correspondence, the predicted intervals become narrower, indicating a more reliable relationship between the locations on scalp and in brain. These results imply that if we locate each channel in the same scalp position *s*_*i*_, as defined in CPC, across participants, that is,

(5) $$s_{i1}=s_{i2}=\ldots =s_{iN_{p}}=s_{i},\;i\in \left\{1,2,\ldots ,N_{c}\right\},$$

then the measured brain locations will have lower variability. The variability of brain locations is given by the TBA (to about 5 mm accuracy as shown in Figure 1) as:

(6) $${\hat{\mathrm{GIV}}}_{i}=\sigma \left(B_{s_{i}}\right),$$

where $B_{s_{i}}$ is the variable indicating brain locations of participants in the population (*N* = 114) corresponding to scalp location *s*_*i*_, and $\sigma \left(B_{s_{i}}\right)$ is the variability of these brain locations given by the TBA.

#### 2.2.2 Optimization of GIA using TBA

Assuming the channels can be located at the same scalp location *s*_*i*_ across participants, the remaining question is how to determine *s*_*i*_ to optimize GIA using a TBA. The GIA_*i*_ value can be approximated using data from the TBA:

(7) $${\hat{\mathrm{GIA}}}_{i}=P\left(l|s_{i}\right),\;l\in \mathrm{ROI},$$

where *P*(*l*|*s*_*i*_) is the probability of scalp location *s*_*i*_ hitting the target brain area *l* for a population given by the TBA. Therefore, $\hat{\mathrm{GIA}}$ can be optimized by arranging the channels at scalp locations with high *P*(*l*| *s*_*i*_), that is,

(8) $$\left(s_{1}^{*},s_{2}^{*},\ldots ,s_{N_{c}}^{*}\right)=\underset{\left\{s_{1},s_{2},\ldots ,s_{N_{c}}\right\}}{\text{argmax}}\frac{1}{N_{c}}\sum_{i=1}^{N_{c}}{\hat{\mathrm{GIA}}}_{i}=\underset{\left\{s_{1},s_{2},\ldots ,s_{N_{c}}\right\}}{\text{argmax}}\frac{1}{N_{c}}\sum_{i=1}^{N_{c}}P\left(l|s_{i}\right).$$

Here, vector $\left(s_{1}^{*},s_{2}^{*},\ldots ,s_{N_{c}}^{*}\right)$ holds the channel locations which have a maximum $\hat{\mathrm{GIA}}$ among the possible optode arrangements given by an optode montage.

### 2.3 Algorithm

In the following section, based on the above proposed objective functions, an optimization algorithm for automatically optimizing $\hat{\mathrm{GIA}}$ on a typical scalp in virtual space is presented. We first detail several inputs for defining the automatic optode arrangement problem. Then, the optimization procedure is illustrated. Finally, the inputs and outputs of the automatic optode arrangement algorithm are summarized. The placement procedure for the resultant montage is also illustrated, including making the optode montage in physical space and placing it on each participant's scalp while guided by a navigation system, which can reduce scalp location variability across participants.

#### 2.3.1 Automatic optode arrangement on virtual scalp

##### Typical scalp

**FIGURE 2:**
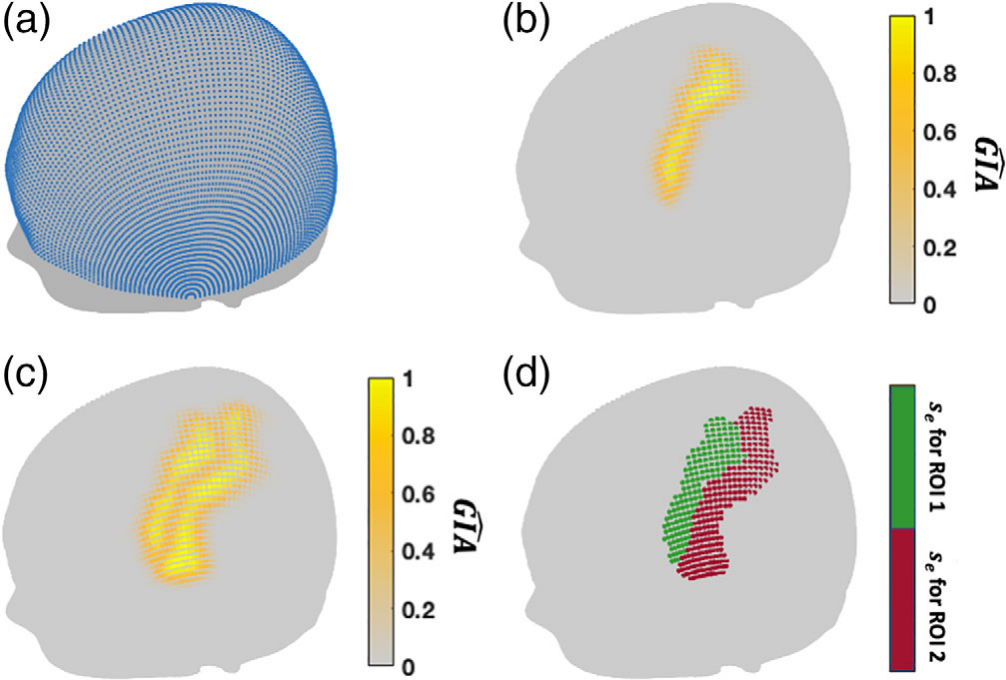
(a) CPC100 (blue dots) measured on the surface of the Chinese2020 scalp template. (b) ${\hat{\mathrm{GIA}}}_{s^{*}}$ map of precentral gyrus given by transcranial brain atlas (TBA) (automatic anatomical labeling [AAL]) and displayed on the scalp surface. (c) ${\hat{\mathrm{GIA}}}_{s^{*}}$ map of precentral and postcentral gyri. (d) Effective scalp locations (${\hat{\mathrm{GIA}}}_{s^{*}}>0.5$) for the precentral (green) and postcentral (red) gyri

A typical scalp in virtual space needs to be prepared to enable automatic arrangement of optodes. The typical scalp should be able to represent the shape and size of the group of participants to be imaged, to ensure that for most of the participants in the group, the optode montage can be placed close to the locations transferred from the optimized arrangement. The CPC100 is then automatically measured on the segmented scalp of the head template (Figure 2a). For ∀s^*^ ∈ *CPC*100, where s^*^ denotes the CPCs on the typical scalp, the *P*(*l*| s^*^), that is, ${\hat{\mathrm{GIA}}}_{s^{*}}$, can be obtained from the selected TBA.

##### Regions of interest

Based on the aim of the study, users can choose ROIs from previously constructed TBAs, including anatomical parcellations such as BA and AAL, or functional TBAs derived from meta‐analysis. As an example, a ${\hat{\mathrm{GIA}}}_{s^{*}}$ map of precentral gyrus given by AAL‐based TBA is displayed on the typical scalp (Figure 2b). Note multiple parcellations can be chosen for automatic arrangement. When the ROIs are adjacent to each other, the ${\hat{\mathrm{GIA}}}_{s^{*}}$ is the value at the location with the maximum probability of hitting the selected ROIs (Figure 2c), as follows:

(9) $${\hat{\mathrm{GIA}}}_{s^{*}}=\mathrm{Max}\left(P\left(l_{i}|s^{*}\right)\right),\;l_{i}\in \text{ROIs},$$

where *i* is the index of the selected ROIs. When ROIs are separated, the optodes can be arranged for each ROI in sequence. Users can also construct their own TBAs based on the aims of a study, using the method published previously (Jiang et al., 2020).

As mentioned in Section 2.2.2, the channels should be arranged at the scalp locations with high $\hat{\mathrm{GIA}}$, that is, ${\hat{\mathrm{GIA}}}_{s}>t$. The scalp locations with ${\hat{\mathrm{GIA}}}_{s}>t$ can be defined as effective scalp locations *s*_*e*_, for some *t* defined by user (Figure 2d). Given the possible channels formed by the optode arrangement, the channels placed at ${\hat{\mathrm{GIA}}}_{s}>t$ locations are defined as the effective channels (number of them denoted by *N*_*ce*_). In this study, we suggest *t* = 0.5, since when *t* < 0.5, the channel would cover the ROI in less than half of the participants, which can be considered as unreliable for channel‐wise analysis. Note that in some cases there is a tradeoff between the number of effective channels and the average $\hat{\mathrm{GIA}}$. Since the number of effective channels can only be slightly different under different optode arrangements due to spatial constraints, we let the algorithm maximize the average $\hat{\mathrm{GIA}}$ of channels produced by the optode arrangement under each possible value of *N*_*ce*_. The algorithm will output the optode arrangement with maximum $\hat{\mathrm{GIA}}$ for each number of effective channels, then the user can choose to prioritize average $\hat{\mathrm{GIA}}$ or number of effective channels based on the situation at hand.

##### Other constraints

**FIGURE 3:**
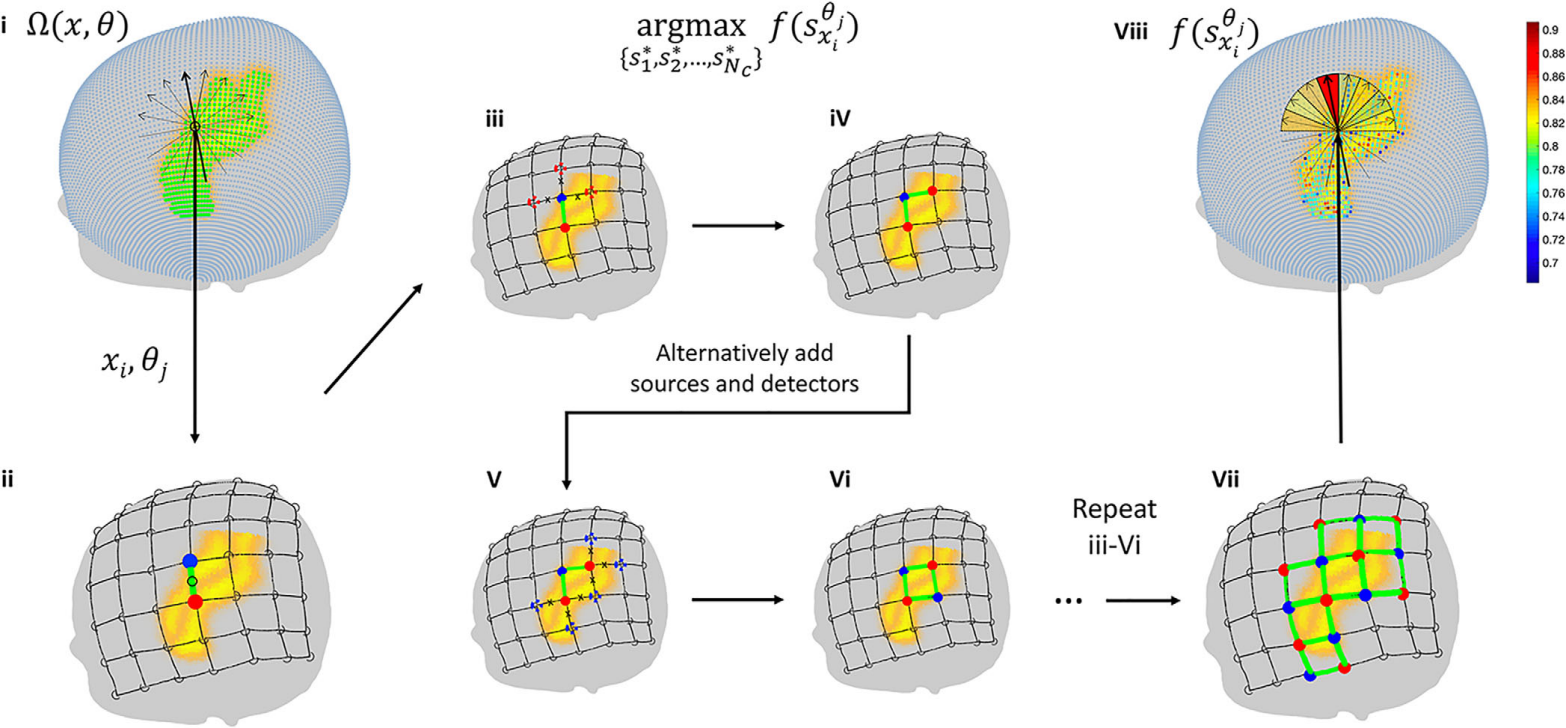
Flowchart of the optimization procedure. (i) Parameter space Ω(*x*, *θ*) of spatial constraints, which includes centers (green dots) and orientations (black arrows). (ii–vii) Optode arrangement procedure under one spatial constraint *x*_*i*_, *θ*_*j*_ (black grid). A seed channel (green line) formed by a source (blue dot) and a detector (red dot) is first aligned to the center of the grid (iii). Sources and detectors are alternatively added to the locations around the placed optodes to maximize the objective function (iii–vi) until no more optodes remain (vii). The value of the objective function $f\left(s_{x_{i}}^{\theta_{j}}\right)$ is mapped to the parameter space Ω(*x*, *θ*)

For topographic fNIRS imaging, the optodes are often fixed by holders, which incorporate certain spatial constraints that should be considered by the automatic optimization procedure. For example, manufacturers such as Hitachi and Shimadzu provide holders in a grid shape with fixed source‐detector distance. Here we considered grid montages supported by the majority of topographic fNIRS instruments, which are implemented according to Tsuzuki et al. (2007). As a montage can be placed at any scalp location with arbitrary orientation, the space of optode montage placement has parameters of center and orientation, denoted Ω(*x*, *θ*) (Figure 3i). Note that once the grids are placed on the virtual typical scalp, the resultant optode and channel locations (in CPCs), as well as their neighborhood relations, can be saved by our software, which allows reuse by the automatic optimization algorithm. Using parallel computing, it took about 1 day to analyze all montage placements (here we discretized arrangement space to 500(*x*) × 30(*θ*)) for the two ROIs used in the current study, using an HP tower server with quad‐core processors (Intel Xeon E5‐2407 and 2403).

The user should also input the optode count, including numbers of sources and detectors, to be arranged. Note that this step is optional, since the algorithm can automatically decide the number of optodes needed to cover the ROIs.

##### Optimization procedure

The optimization procedure for optode arrangement is shown in Figure 3. Theoretically, the center of the probe montage can be placed at any scalp location with arbitrary orientation. For each center location and orientation, optodes should be arranged to maximize average $\hat{\mathrm{GIA}}$. Therefore, the optimization algorithm contains two levels. In the first level, the average $\hat{\mathrm{GIA}}$ is maximized using a greedy algorithm for each of *x*_*i*_ and *θ*_*j*_ (Figure 3ii–vii). Specifically, the seed channel is first located at *x*_*i*_ by placing a pair of optodes with orientation *θ*_*j*_. Then, a source (or detector) is added to the position around the existing optodes where a channel with maximum $\hat{\mathrm{GIA}}$ can be generated. The sources and detectors are alternatively added until no more effective channels can be generated. Any excess optodes are randomly placed around the existing optodes; they can form control channels. If the user specifies the numbers of sources and detectors, the algorithm stops when all the available optodes are consumed. In the second level, optode arrangements with maximum average $\hat{\mathrm{GIA}}$ are selected for each value of the number of effective channels. Note that in real‐world situations, the optode montage is often arranged at scalp locations corresponding to ROIs. Therefore, by default, only effective scalp locations are searched, that is, *x*_*i*_ ∈ *s*_*e*_. For large ROIs, the center of the optode montage is often placed at a location near the center of the ROI, which can be derived by $\hat{\mathrm{GIA}}$‐weighted center of mass, that is, $s_{e}^{c}=\sum_{i\in s_{e}}\frac{s_{i}∙{\hat{\mathrm{GIA}}}_{i}}{{\hat{\mathrm{GIA}}}_{i}}$. Therefore, users can also optionally select a range from the center of ROIs to limit the *x* to be searched. The orientation *θ*_*j*_ is implemented as $\theta_{j}\in \left\{0,\frac{\pi }{n_{\theta }},\frac{2\pi }{n_{\theta }},\ldots ,\pi \right\}$ where *n*_*θ*_ is the resolution of the search on orientation.

##### Summary of user inputs and outputs

To clarify the usage of the TBA‐based automatic optode arrangement algorithm, the user‐inputs to the algorithm are summarized as follows:





*ROIs*;


*Threshold for effective scalp locations (t)*;


*Representative scalp*;


*Spatial constraints imposed by the optode holder*;


*Number of sources and detectors available (nS*, *nD)*;


*Range of optode montage parameter space (**x*, *n*_*θ*_*)*.






After input of the parameters and execution of the automatic optode arrangement algorithm, the results can be visualized (Figure 5). The montage placement results, including locations of both optodes and channels, are presented along with the ${\hat{\mathrm{GIA}}}_{s^{*}}$ map on the typical scalp. This enables users to decide how to physically make the optode holder following the placement results. Users can discard the optodes which form ineffective channels to accelerate the process of installing optodes.

#### 2.3.2 Optode montage placement in physical space

A head cap can be prepared to facilitate placing the optode montage on the scalps of participants. The head cap is first placed on a physical scalp, which can either be a 3D‐printed model or on a real person. The optode positions can then be marked on the head cap guided by a navigation system. Finally, the optode montage can be aligned to the markers and fixed on the head cap. For optode holders equipped with bandages to affix to the head of participants, the optode holder can be directly placed for each participant following the montage placement procedure.

**FIGURE 4:**
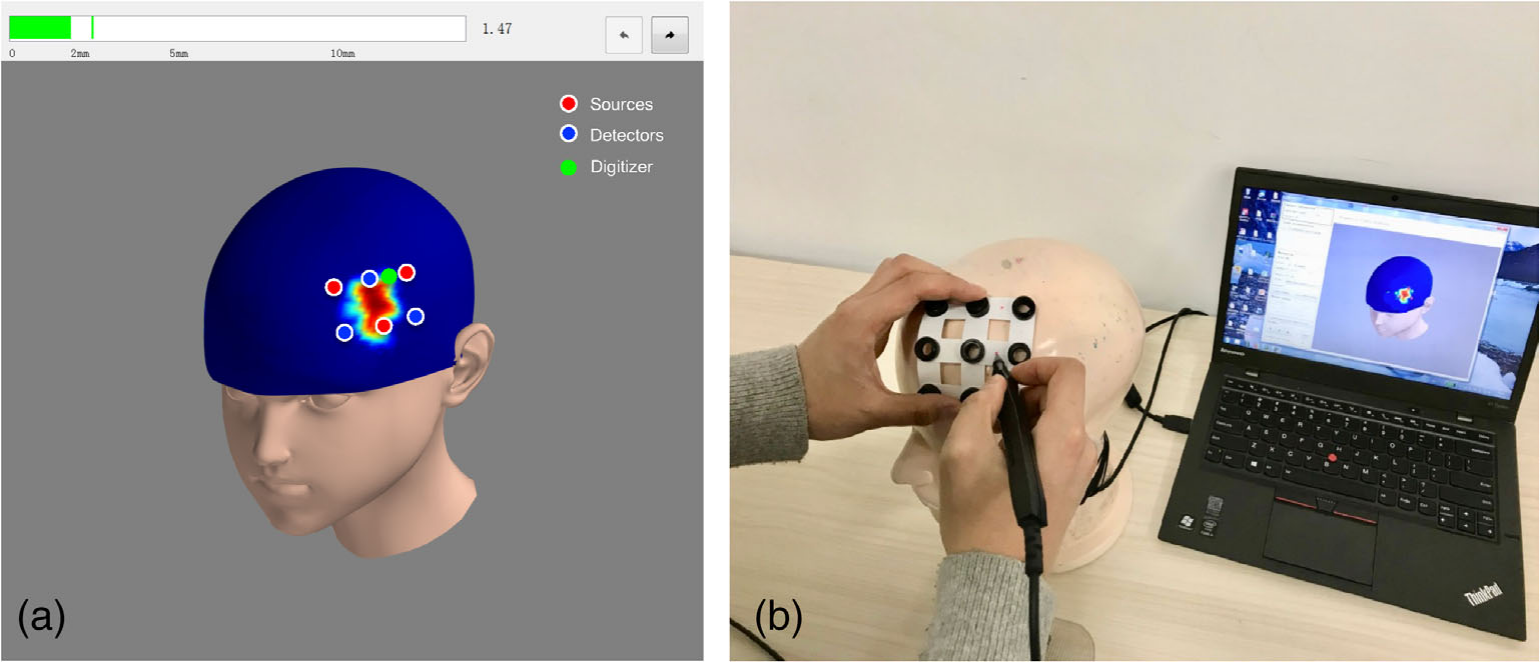
Optode montage placement on a participant's scalp guided by a scalp navigation system. (a) GUI of the navigation system. Automatically arranged optodes are displayed on a virtual head model. (b) Optode montage placement on a physical head model guided by the digitizer

To ensure the accurate placement of the optode montage on each participant, instead of visually aligning the head cap, we use a custom‐made navigation system to facilitate the placement of the head cap (Figure 4). The scalp navigation system enables the alignment of each channel on the physical optode montage with the location decided in the automatic arrangement step. The placement procedure is as follows. First, we fix the small transmitter of the 3D‐digitizer on the participant's face using a wound plaster. Second, we perform sparse sampling and scalp reconstruction to link physical scalp space to virtual scalp space, as visualized in the navigation system (Figure S2). Third, the head cap is placed on the participant's head. Fourth, we slightly adjusted the head cap so that the channels are aligned to the channels on screen as closely as possible. Note that when the optode montage is rigid, it is impossible to perfectly align all channels between participants, as their head sizes and shapes are most likely different. Therefore, we suggest prioritizing the accuracy of effective channels since they are more important to a study. A more detailed description of the usage of the navigation system can be found in the Supplementary Material.

### 2.4 Application experiments

To validate the proposed method and test whether it can outperform the widely used 10/20 optode arrangement method, we arranged and placed optode montages for a group of participants with the goal of studying two brain functions. Motor (finger‐tapping) and working memory (n‐back) functions were selected as targets, since they are well‐studied and cover both low‐ and high‐level cognitive systems.

#### 2.4.1 Optode arrangement and placement

The input parameters for optode arrangement in the application experiment are listed in Table 1. To target motor function, precentral and postcentral gyri of the AAL anatomical TBA were selected as ROIs. The centers of optodes montages to be searched were set as those locations within four CPC units of the center of the ${\hat{\mathrm{GIA}}}_{s^{*}}$ map, which is derived by calculating the $\hat{\mathrm{GIA}}$‐weighted center of mass. For working memory function, a working memory functional TBA was used (Jiang et al., 2020). For both tasks, since a LABNIRS system (Shimadzu) was used, the spatial constraint is a montage with square shape and 3 cm source‐detector distance. The Chinese2020 scalp template (http://www.chinese-brain-atlases.org/download.html) was used as the virtual scalp, since our study participants were Chinese. Based on the outputs of the automatic optode arrangement algorithm, a head cap was produced and placed following the procedure described in Section 2.3.2.

**TABLE 1:** Input parameters of the automatic optode arrangement algorithm

ROIs	*t*	Typical scalp	Spatial constraints	*nS*, *nD*	*x*, *n*_*θ*_
Precentral and postcentral gyrus, AAL, anatomical TBA	0.5	Chinese2020	Square‐shape, source‐detector distance 3 cm	8, 7	$\left|s_{e}^{c}-x\right|<4,$ 30
Working memory function, functional TBA	0.5	Chinese2020	Square‐shape, source‐detector distance 3 cm	3, 3	*s*_*e*_, 30

Abbreviations: AAL, automatic anatomical labeling; ROI, region of interest; TBA, transcranial brain atlas.

The 10/20‐based optode arrangement and placement were done as follows. In the arrangement step, a swimming cap was first fixed on a 3D‐printed head model (Chinese2020). The 10/20 landmarks were manually measured and marked on the cap. Then, the montage was fixed to the swimming cap by aligning to the 10/20 landmarks suggested by the literature (Jasper, 1958; Okamoto et al., 2004). Specifically, for motor function, a 3 × 5 rectangular montage (same number of sources and detectors as in the TBA‐based method) was fixed on the head cap by aligning the midline of the montage with the T3‐C3‐Cz line on the head cap and aligning C3 with the center optode on the montage (Figure 6). For working memory function, a 3 × 2 rectangular montage was placed so that the center of the montage was aligned with F4 and the bottom line of the optode montage was parallel to the eyebrow (Figure 6). In the montage placement step, the head cap was placed on each participant by visually aligning the midline of the optode montage with the midline of the participant's head for the motor task. For working memory, the head cap was placed on the participant by visually aligning the rim of the head cap with the eyebrow of each participant.

#### 2.4.2 Experimental paradigm

Nine participants were recruited to perform both motor (finger‐tapping) and working memory (n‐back) tasks. Their structural MRI (T1‐weighted) images were obtained in a previous experiment. Before the experiment, informed consent was obtained from each participant. In the finger‐tapping task, participants alternatively pressed their index and ring fingers on a keyboard. The task consisted of eight blocks of 15‐s duration followed by a 15‐s rest for each block. In the n‐back task, participants were tested under three conditions of the verbal n‐back paradigm, 0‐back, 1‐back, and 2‐back, in pseudorandomized order, and each condition consisted of three blocks. Randomly chosen letters (among “A,” “B,” “C,” “D,” “E,” “F,” “G,” “H,” “J,” and “L”) were presented in quick succession on a computer screen with a black background. Participants were instructed to respond to every stimulus, indicating whether the current letter was the same as the target letter. The target letter was the current, preceding first or preceding second letter, for the 0, 1 or 2‐back conditions, respectively. Each letter appeared for 300 ms, with an interstimulus interval of 1,700 ms. Each participant performed a total of nine blocks, which included three blocks for each condition. Each block contained a 22‐s period of rest followed by a task period of 24 s. A Shimadzu LABNIRS fNIRS recording system was used for recording the brain activity of all participants.

#### 2.4.3 Data analysis

The measurement locations of the placed montage were evaluated based on individual scalp‐brain correspondences provided by individual sMRI images. Specifically, for each participant, the coordinates as well as labels in common brain space, that is, MNI space, for evaluating GIV and GIA were obtained as follows. First, locations of optodes were registered to sMRI space based on four fiducial markers (Nasion, Inion, left preauricular, and right preauricular) using rigid transformations. The fiducial markers were digitized on the participant's head using a 3D digitizer in physical space and visually identified in sMRI space. Second, the individual sMRI images were segmented using SIMNIBS (Thielscher, Antunes, & Saturnino, 2015), and channel locations were projected to the segmented gray matter surface using the balloon‐inflation model (Okamoto & Dan, 2005). Finally, the channel locations in MNI space were obtained by registering the gray matter positions in sMRI space to MNI space using SPM12. The labels of the channels were obtained using the AAL anatomical brain atlas and functional brain atlas made by Jiang et al., in MNI space for the finger‐tapping task and the n‐back task, respectively (Jiang et al., 2020; Rolls, Joliot, & Tzourio‐Mazoyer, 2015; Tzourio‐Mazoyer et al., 2002).

Functional data of both the finger‐tapping and n‐back tasks were analyzed using NIRSPM software (Ye, Tak, Jang, Jung, & Jang, 2009). To make comparison of optode arrangement methods independent of preprocessing methods, default preprocessing parameters were used, which included a detrending with DCT‐based high‐pass filter (128 s) and low‐pass filtering with the HRF. To compare the group activation results, we estimated the effect size ($\hat{\beta }$) of ∆HbO_2_ for both methods and tasks. In the finger‐tapping task, we chose the contrast of task > rest. In the n‐back task, we chose 1 and 2 back >0 back as the contrast. The group results were derived by averaging individual beta values in a channel‐wise manner.

## 3 RESULTS

### 3.1 Optode arrangement results

**FIGURE 5:**
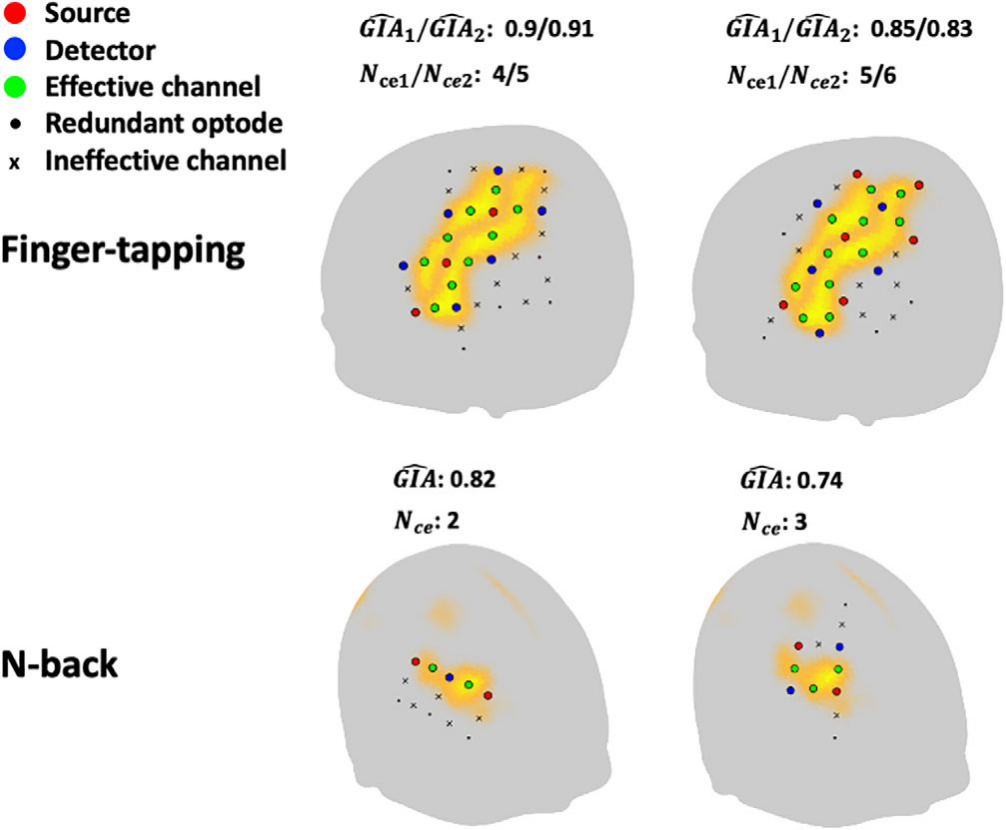
Representative automatically‐found arrangements on a virtual typical scalp (Chinese 2020 scalp template)

Two representative results of the optode arrangement procedure for each task are shown in Figure 5. For both tasks, it can be seen that the average $\hat{\mathrm{GIA}}$ decreases with an increase in the number of effective channels. As mentioned in Section 2.3.1, we prioritize average $\hat{\mathrm{GIA}}$ rather than number of effective channels. Therefore, we selected optode arrangements with numbers of effective channels *N*_ce1_ = 4 and *N*_ce2_ = 5 for the finger‐tapping task and *N*_*ce*_ = 2 for the n‐back task. For both tasks, the computational cost was less than 10 min.

### 3.2 Comparison with traditional 10/20‐based method

#### 3.2.1 Optode arrangement and placement results

**FIGURE 6:**
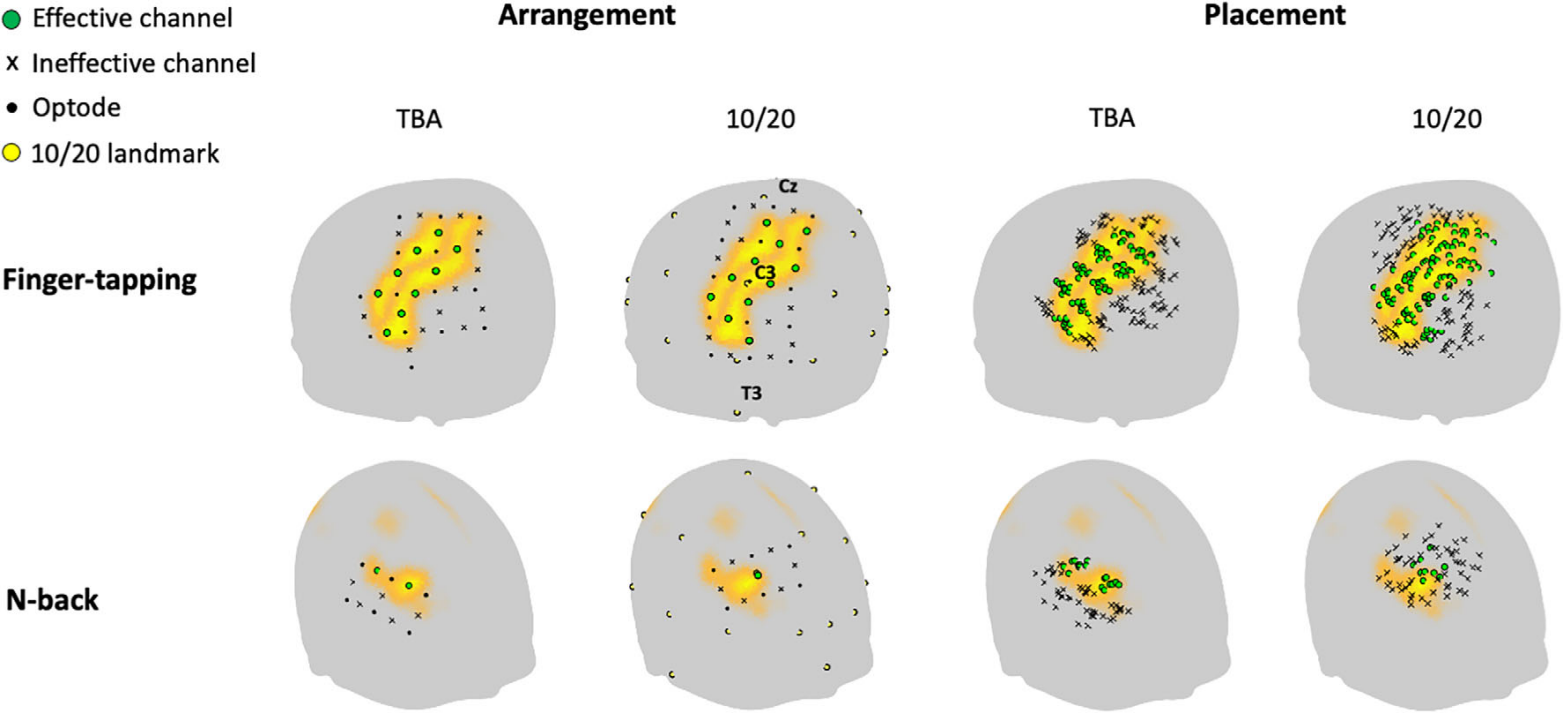
Comparison of transcranial brain atlas (TBA)‐ and 10/20‐based optode arrangement and placement results

A comparison of TBA‐based and 10/20‐based optode arrangement is shown in Figure 6. Although the montage arrangement results show that both methods produced effective channels covering the ROIs, it can be seen that the channels were located on scalp locations with higher $\hat{\mathrm{GIA}}$ for the TBA‐based method compared to the traditional 10/20‐based method. Quantitatively, the TBA‐based method had an average $\hat{\mathrm{GIA}}$ of 0.9 and 0.91 for precentral and postcentral gyrus (finger‐tapping) and 0.82 for DLPFC (n‐back), whereas the 10/20‐based method had an average $\hat{\mathrm{GIA}}$ of 0.72 and 0.7 for precentral and postcentral gyrus and 0.68 for DLPFC. Since we prioritize the average $\hat{\mathrm{GIA}}$ in the TBA‐based arrangement, the 10/20‐based method had more effective channels (*N*_ce1_ = 5 and *N*_ce2_ = 6) for the finger‐tapping task. However, compared with the average $\hat{\mathrm{GIA}}$ produced by the TBA‐based arrangement with the same number of effective channels (0.85 for ROI1 and 0.83 for ROI2), the 10/20‐based method also had lower average $\hat{\mathrm{GIA}}$. Therefore, the TBA‐based method produced more optimized optode arrangements than the traditional 10/20‐based method in general.

The consistency of channel locations on the scalp was higher for the navigation‐based placement used in the TBA‐based method. The values of channel scalp variability for both methods are presented in Table 2. The values indicate an increase in consistency of placed locations when using navigated placement.

**TABLE 2:** Scalp location variability (mm)

ROI	TBA	10/20
Finger‐tapping	4.66 ± 0.70	6.93 ± 1.07
n‐Back	5.27 ± 0.56	9.56 ± 0.19

Abbreviations: ROI, region of interest; TBA, transcranial brain atlas.

#### 3.2.2 Activation results

**FIGURE 7:**
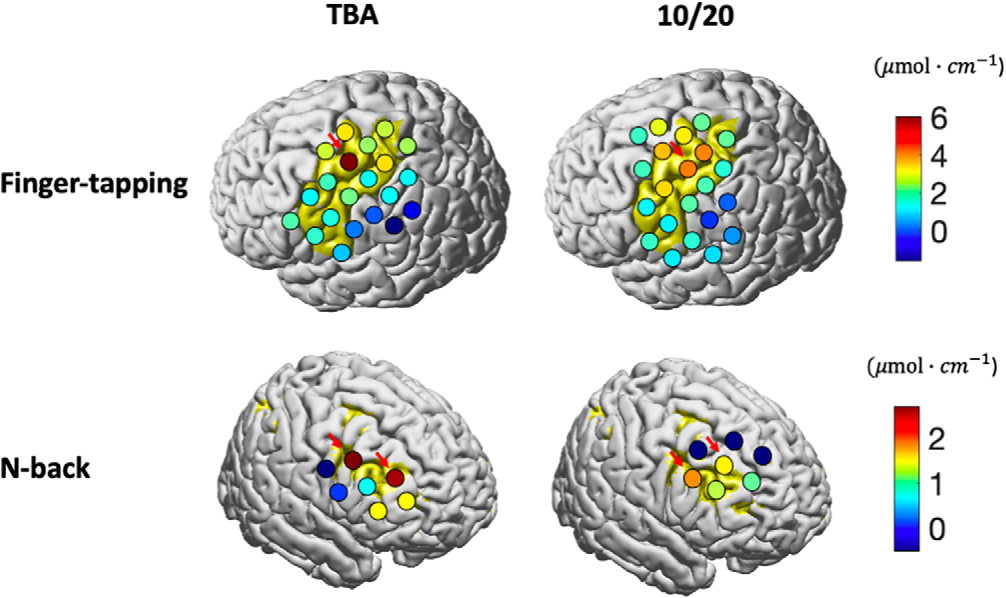
Comparison of task activations measured by montages placed using transcranial brain atlas (TBA)‐based or 10/20‐based methods. Beta values are depicted on the cortical surface of the Colin 27 brain template. Red arrows point to effective channels depicting stronger effects from the TBA‐based method compared to the 10/20‐based method

The activation results further support the claim that our proposed method outperforms the traditional 10/20‐based method. In the finger‐tapping task, the channel with maximum effect size ($\hat{\beta }$) is located in the hand knob area, covered by the TBA‐based optode arrangement method (Figure 7). The activation map produced by the 10/20‐based method is relatively more dispersed and weaker. In the n‐back task, results show the same trend as in the finger‐tapping task. In all, compared with the 10/20‐based method, activation results produced by the TBA‐based method are more reliable and are in accordance with the literature.

#### 3.2.3 GIV and GIA

The better activation results can be attributed to improved optode arrangement quality as well as placement quality. The evaluation indices of effective channels were derived using Equations (1), (2), (3), (4)) based on individual sMRI images (Table 3). The results show a lower average GIV for both motor (7.26 vs. 7.76 mm) and working memory (8.07 vs. 10.51 mm) when placing via the TBA‐based method. The proposed method also had a higher average GIA for both motor areas (0.74 vs. 0.67) and DLPFC (0.67 vs. 0.56). Note, no *SD* is given for the n‐back task and 10/20, since only one channel was effective. These results support the claim that the proposed method outperforms the 10/20‐based method in optode placement quality.

**TABLE 3:** GIA and GIV of effective channels in brain space

Indices	ROI	TBA (mean ± *SD*)	10/20 (mean ± *SD*)
GIA	Finger‐tapping	0.74 ± 0.13	0.67 ± 0.13
	n‐Back	0.67 ± 0.16	0.56
GIV (mm)	Finger‐tapping	7.26 ± 0.72	7.76 ± 0.95
	n‐Back	8.07 ± 0.22	10.51

Abbreviations: GIA, group imaging accuracy; GIV, group imaging variability; ROI, region of interest; TBA, transcranial brain atlas.

To further investigate the differences in the activation patterns obtained by the TBA‐based method compared to the 10/20‐based method, we focus on channels with stronger activation effects in the activation map produced by TBA‐based method. We compare their placement quality with that of channels spatially closed to their brain locations in montages made by the 10/20‐based method (pointed to by red arrows in Figure. 7). In the finger‐tapping task, the TBA‐based method obtained a higher GIA (1 vs. 0.67), but a higher GIV (7.57 vs. 6.88 mm) for this channel. Therefore, we believe the stronger activation is produced by the increased GIA of this channel. In the n‐back task, since the TBA‐based method had both higher GIA (0.67 vs. 0.50 on average) and lower GIV (8.07 vs. 10.70 mm on average) for these channels compared to the 10/20‐based method, the better activation map is likely due to improved performance of both GIA and GIV.

## 4 DISCUSSION

In this study, we established the theoretical basis of TBA‐based optimization of fNIRS optode arrangement. Based on this theoretical basis, we proposed a two‐step procedure, consisting of automatic arrangement and navigation‐assisted placement. The results of our validation experiments indicate our method outperforms the traditional 10/20‐based method in both optode arrangement quality and task activation results.

We set our comparison target as the traditional 10/20‐based method because it is the most widely used method in the current field of topographic fNIRS. There are also other methods supporting optode arrangement for topographic fNIRS. Cutini's method requires a 3D‐printed head model, manual operation, as well as an optical navigation system, which may be inaccessible for many laboratories. Although Morais's approach does not need external devices and incorporates brain atlases, the optodes can only be arranged at a relatively restricted set of scalp locations, namely the 10/20, 10/10 or 10/5 system locations, which can only be used with some fNIRS recording systems. Therefore, to the best of our knowledge, the 10/20‐based method is still the most widely used optodes arrangement method in the fNIRS community.

From the results of optode arrangement indices derived using individual sMRI of participants (Table 3), it can be seen that the actual GIA and GIV deviate from the estimated $\hat{\mathrm{GIA}}$ and $\hat{\mathrm{GIV}}$ given in the optode arrangement procedure, as mentioned in Section 3.2.1. Theoretically, the optode arrangement (Step 1) determines the GIA and GIV by deciding the scalp locations of channels. However, deviations originate from both parts of the optode arrangement and placement procedure. The first part is the usage of scalp‐brain correspondences derived from a population instead of the participants to be imaged. The second part is inconsistent placement, which adds error when adjusting rigid probe holders to participants' heads, which vary in shape and size, as well as when manually placing optode montages (here, we do not distinguish between these two sources of error). This can be mathematically expressed as $\mathrm{GIA}=\hat{\mathrm{GIA}}+\varepsilon_{1}^{A}+\varepsilon_{2}^{A}$ and $\mathrm{GIV}=\hat{\mathrm{GIV}}+\varepsilon_{1}^{V}+\varepsilon_{2}^{V}$, where *ε*_1_ and *ε*_2_ represent the error introduced from using substitutive scalp‐brain correspondence and from inconsistent placement, respectively. *ε*_1_ and *ε*_2_ can be evaluated using individual sMRI images of the participants. Specifically, $\varepsilon_{1}^{A}$ can be estimated as the difference between $\hat{\mathrm{GIA}}$ given by the TBA and by individual sMRI images, and $\varepsilon_{2}^{A}$ can be derived by subtracting $\hat{\mathrm{GIA}}+\varepsilon_{1}^{A}$, which is the GIA when no placement error is involved, from the actual GIA. $\varepsilon_{1}^{V}$ and $\varepsilon_{1}^{V}$ can be derived using the same manner. We applied the above analysis procedure to the effective channel locations in both tasks of our experiment. The results indicate that $\varepsilon_{1}^{A}$ and $\varepsilon_{2}^{A}$ are 0.19 ± 0.16 (mean ± *SD*) and 0.26 ± 0.18, respectively; $\varepsilon_{1}^{V}$ and $\varepsilon_{2}^{V}$ are 1.53 ± 0.99 mm and 2.68 ± 1.17 mm, respectively. It can be seen that both parts contributed to the deviation of the actual GIA and GIV from $\hat{\mathrm{GIA}}$ and $\hat{\mathrm{GIV}}$. The error introduced by inaccurate placement is also higher than that from using substitute scalp‐brain correspondence. To minimize *ε*_1_, one can incorporate populational features, such as age, gender, and so forth, to obtain a more accurate scalp‐brain correspondence for the participant to be imaged. *ε*_2_ can be minimized by using more advanced techniques during placement of the optode montage. In this study, we used navigation‐assisted placement for the TBA‐based optode arrangement to reduce *ε*_2_. Other techniques have been proposed for assisting optode montage placement. For example, Kawaguchi and Yamada used an augmented reality technique, which results in positioning accuracy similar to our method (Kawaguchi & Yamada, 2019). Another part of *ε*_2_ originates from using rigid optode montages (while participants have head size and shape differences). An easy way to reduce this error is to select participants with similar head size and shape. The head size and shape information can be evaluated before imaging a participant using the head‐shape digitizing system. Thus, a participant with outlier head size and shape can be screened out, so as to save valuable experimental resources. Also, *ε*_1_ and *ε*_2_ can both be reduced by increasing the number of participants to be imaged, as shown in the predicted intervals of the curve fitting the relationship between variability in scalp and brain space (Figure 1). This is also a limitation of the current study, in which only nine participants were recruited.

To further investigate the actual GIA and GIV are mainly influenced by which step in our experiment (optode arrangement or placement), we performed a correlation analysis between the actual GIA and $\hat{\mathrm{GIA}}$ and $\varepsilon_{2}^{A}$, and the actual GIV and $\hat{\mathrm{GIV}}$ and $\varepsilon_{2}^{V}$, respectively. The results indicate that GIA is significantly correlated to $\hat{\mathrm{GIA}}$ instead of $\varepsilon_{2}^{A}$ (*r* = .60, *p* < .01), while GIV is significantly correlated to $\varepsilon_{2}^{V}$ instead of $\hat{\mathrm{GIV}}$ (*r* = .68, *p* < .001). Since $\hat{\mathrm{GIA}}$ and $\hat{\mathrm{GIV}}$ are set in the optode arrangement step, while $\varepsilon_{2}^{A}$ and $\varepsilon_{2}^{V}$ are set in the optode placement step, these results indicated that the actual GIA and GIV are mainly influenced by optode arrangement and placement, respectively. In the finger‐tapping task, since the stronger activation from our method is mainly caused by increased GIA as discussed in the activation results section, we believe that the optode arrangement is the main reason for better activation detection. In the n‐back task, since both GIA and GIV are better for our method, we suggest that both optode arrangement and placement contributed to the better activation detection.

In the n‐back task, we can see the variability in placed scalp locations; GIV is reduced less by the navigation‐assisted placement (versus manual placement) in the finger‐tapping task (Table 3). It seems that the navigation‐assisted placement is more useful for the n‐back task compared to the finger‐tapping task, which can be attributed to two reasons. First, a smaller optode montage is used in the n‐back task compared to the finger‐tapping task. Since the optode montage is rigid, a larger optode montage will generate larger scalp variability in channel locations due to head shape and size variation across participants, especially for channels on the margin of the optode montage, which has been discussed by Cutini et al. (2011). Second, in the finger‐tapping task, we placed the 3 × 5 optode montage by aligning the midline of the optode montage with the Cz‐C3‐T3 line in the 10/20‐based optode placement. These 10/20 locations are relatively easy to identify since their locations are directly related to the fiducial points, for example, T3 is above AL, and Cz is in the middle of the Nz to Iz line. However, for the n‐back task, F4, is harder to find since its location is based on the scalp locations of other 10/20 landmarks. Previous studies also found differences between 10/20 points in terms of measurement error for, and suggested that the measurement error of F4 is larger than that of T3, C3, and Cz (Sparing, Buelte, Meister, Pauš, & Fink, 2008; Xiao et al., 2017). Therefore, we suggest that navigation is more useful for placing small optode montages at scalp locations where no 10/20 landmarks are not easily measured.

**FIGURE 8:**
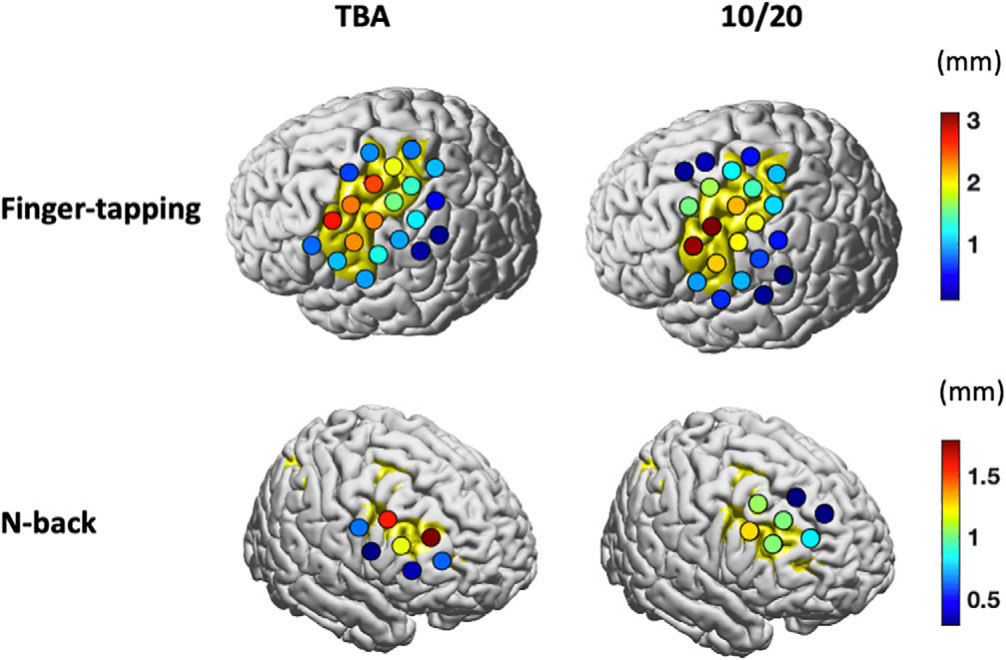
Comparison of the sensitivity produced by transcranial brain atlas (TBA)‐based and 10/20‐based optode arrangement methods

In this study, the indices for optimization of optode arrangement quality are defined based on a simplified projection‐based model, which assumes the measurement location is the cortical point projected from the channel location on the scalp. This general model is also used for localization of other transcranial techniques like TMS and EEG. In a realistic fNIRS model, the measurement locations of a pair of optodes are the brain locations traversed by the photons, often termed the banana‐shaped optical path, from source to detector. Therefore, one may question whether an optimization based on a simplified model can lead to increased performance in optode placement quality evaluated based on a realistic fNIRS model. Thus, we compared optode placement quality of our method with the 10/20‐based method using a realistic fNIRS model (methods are described in the Supplementary Material). The results show that the sensitivity of effective channels produced by the TBA‐based method are more uniform and also more focal to ROIs than those from the 10/20‐based method (Figure 8). In other words, our method also achieves higher performance when the measurement locations are evaluated using a realistic fNIRS model.

Besides the theoretical advances and the proposed method for fNIRS optode placement, the current study makes the following contributions. First, we experimentally demonstrated the relationship between the variability in scalp space and brain space and its reliability for different numbers of participants. This is fundamental for optode alignment in fNIRS, and also other transcranial brain imaging or stimulation techniques when participant specific sMRI images are unavailable. Second, the proposed method can be directly applied to other transcranial techniques, such as EEG, TMS, or TDCS, for optimizing placement of recording or stimulating elements. Third, since GIA and GIV are important for evaluating optode arrangement quality, future fNIRS studies may incorporate these indices in reports, which will help improve interstudy comparison and facilitate interpretation of fNIRS results.

In future studies, TBA‐based optode arrangement can be extend in two directions. First is the construction of fNIRS specific TBAs which incorporates photon propagation simulation at each scalp location for head data from a large sample of people. Using an fNIRS‐specific TBA, optimized scalp locations as well as orientations for placing optodes for a group of participants can be determined. Second, the usage of TBAs should be expanded. Particularly, TBAs should be utilized to support tomographic fNIRS. Machado and Brigadoi have both proposed automatic algorithms for optode arrangement for DOT based on the scalp‐brain correspondences of templates (Brigadoi, Salvagnin, Fischetti, & Cooper, 2018; Machado, Marcotte, Lina, Kobayashi, & Grova, 2014). Whether incorporating TBAs can improve optode arrangement performance for DOT should be evaluated in a future study.

Finally, to make this method more accessible, we hope to build a website where users can input optode arrangement parameters, including number of optodes, ROIs and optode holder patterns, and visualize the results along with 10/20 and 10/10 landmarks. Thus, users can produce head caps by visually examining the relationship of optode locations and cranial landmarks without a navigation system.

## CONFLICT OF INTEREST

The authors declare no conflicts of interest.

## AUTHOR CONTRIBUTIONS

**Yang Zhao**: Conceptualization, Methodology, data curation, formal analysis, writing ‐ original draft. **Xiang Xiao**: Conceptualization, methodology. **Yi‐Han Jiang**: Conceptualization, methodology. **Pei‐Pei Sun**: Data Curation, resources. **Zong Zhang**: Methodology. **Yi‐Long Gong**: Software, resources. **Zheng Li**: Writing ‐ review and editing. **Chao‐Zhe Zhu**: Conceptualization, methodology, supervision, writing ‐ review and editing.

## Supporting information


**Appendix S1** Supporting Information.

**Table S1** Optical properties for segmented tissues.

**Figure S1** The relationship between GIV in scalp space and that in brain space for each 10/20 scalp locations. Red, green, and blue curves represent results derived when using data from *N*_*p*_ = 10, 20, 50 participants, respectively. The dots indicate individual values from each sampled group. The solid line and dash line represent fitted curves and prediction intervals (95%), respectively.

**Figure S2** Scalp reconstruction procedure displayed in the user interface of the navigation system (A‐C) and real‐time localization (D‐F). (A) Digitization results of 4 landmark points includes Nasion (Nz), Inion (Iz), and left/right preauricular points (AL/AR). (B) Sparse sampling of 21 points on the physical head surface. (C) Scalp reconstruction results depicted as yellow dots. The shape of reconstructed scalp is vertically stretched for better visualizing its validity. (D) An arbitrary scalp point *p*_0_ on a physical scalp model is digitized using a 3D digitizer. (E) *p*_0_ is transformed from 3D coordinates into a CPC form. (F) *p*_0_ displayed on the virtual scalp model (black dot).

## Data Availability

The data that support the findings of this study are available from the corresponding author upon reasonable request.
