# Supplementary material for: Transcranial brain atlas‐based optimization for functional near‐infrared spectroscopy optode arrangement: Theory, algorithm, and application
Source: Hum Brain Mapp. 2020 Dec 17;42(6):1657–69. doi: 10.1002/hbm.25318 (PMC7978141; doi:10.1002/hbm.25318)
Supplement: Supplementary file 1 — Appendix S1 Supporting Information. [file HBM-42-1657-s001.docx]

# Supplementary materials

## Evaluation of the relationship between variability in scalp and variability in brain space

A simulation experiment was conducted to evaluate the relationship between variability in scalp space and brain space. To reduce computational cost, scalp locations were generated around 21 10/20 landmarks. The simulation was run based on the sMRI images of the population (N=114) used in [Xiao et al., 2018]. For each 10/20 location, a group of individual sMRI images ($N_{p}$=10, 20, 50) were randomly sampled from the population. Scalp locations were first generated around the 10/20 locations on the scalp of ICBM152 by drawing from Gaussian distributions with 0 mean and different standard deviations, including 0, 1, 2…, 15 (mm), then transferred to the sMRI images of the sampled group using CPCs. Their corresponding brain locations were obtained using individuals’ scalp-brain correspondences, which were derived in a previous study [Xiao et al., 2018]. The GIV values in scalp as well as brain space of the sampled group are evaluated using the equation (1) in the main text. The simulation was repeated 20 times for each 10/20 locations and the fitted curves were derived by fitting the deviations of the sample points using second-degree polynomials.

The results are shown in Figure S1. It can be seen that for all 10/20 scalp locations, the GIV in brain space increases as the variability in scalp space increases. The prediction intervals also become narrower, indicating a more reliable correlation between the variability in scalp and brain space, with an increase in the number of participants used for the calculation.

------------------------------

Insert figure S1 about here

-------------------------------

Figure S1. The relationship between GIV in scalp space and that in brain space for each 10/20 scalp locations. Red, green, and blue curves represent results derived when using data from $N_{p}=10, 20, 50$ participants, respectively. The dots indicate individual values from each sampled group. The solid line and dash line represent fitted curves and prediction intervals (95%), respectively.

## Usage of scalp navigation system

------------------------------

Insert figure S2 about here

-------------------------------

Figure S2. Scalp reconstruction procedure displayed in the user interface of the navigation system (A-C) and real-time localization (D-F). (A) Digitization results of 4 landmark points includes Nasion (Nz), Inion (Iz), and left/right preauricular points (AL/AR). (B) Sparse sampling of 21 points on the physical head surface. (C) Scalp reconstruction results depicted as yellow dots. The shape of reconstructed scalp is vertically stretched for better visualizing its validity. (D) An arbitrary scalp point $p_{0}$ on a physical scalp model is digitized using a 3D digitizer. (E) $p_{0}$ is transformed from 3D coordinates into a CPC form. (F) $p_{0}$ displayed on the virtual scalp model (black dot).

The scalp navigation system consists of a 3D digitizer (FastrakTM, Polhemus) and computer with navigation software written in Python (Fig. S2). The scalp reconstruction procedure contains three steps (Fig. S2 A-C).

First, four fiducial landmarks including, Nz, Iz, Al, AR (Cz is automatically derived from these four landmarks), need to be digitized on the physical scalp (Fig. S2A). These landmarks are registered to the virtual scalp model and the validity of digitization can be checked in the user interface.

Second, we uniformly and sparsely sample on the person’s head shape and run the S3R algorithm to reconstruct the scalp surface, guided by the user interface of the software (Fig. S2B). The validity of the reconstruction results can also be checked visually in our software (Fig. S2C). The real-time localization algorithm can be summarized as follows. Given a 3D point p_0_ (x_r_, y_r_, z_r_) on the participant’s scalp digitized by the 3D digitizer (Fig. S2D), its corresponding 2D CPC is automatically derived in virtual space by calculating its proportional position on the longitude (AL, AR, P’) and latitude (Nz, Iz, Cz) lines (Fig. S2E). As the CPCs are already measured on the virtual scalp model, the corresponding point p_0_’ (x_v_, y_v_, z_v_) can be directly obtained and displayed on the interface (Fig. S2F). Details of the scalp navigation system can be found in our previous publications [Xiao et al., 2018].

## Comparison of sensitivity of channels produced by TBA-based and 10/20-based optode arrangements.

The sensitivity for each channel is evaluated by averaging the sensitivity in channels within ROI across participants as follows:

$$S_{i}=\frac{1}{N_{p}}\sum_{j=1}^{N_{p}} \sum_{v=1}^{N_{v}} {L_{vj}\cdot D}_{vj}$$

$N_{p}$ is the number of participants and $N_{v}$ is the number of voxels. $L_{vj}$ labels whether the voxel v belongs to the ROI. $D_{vj}$ represents photon measurement density in voxel $v$ of participant $j$. To calculate the photon measurement density, the sMRI images (T1-weighted) are first segmented and reconstructed into five tissues, including skin, skull, CSF, grey matter and white matter. Then the optode locations in physical space obtained by a 3D-digitizer were transferred on participant-specific sMRI images as described in section 2.4 (Data analysis) of the main text. The photon measurement density function (PMDF) for each pair of optodes were estimated using Monte Carlo Extreme [Fang and Boas, 2009]. The optical properties assigned to each tissue were given by Strangman et al. [Strangman et al., 2003] and are shown in Table S1. 10^8^ photons were simulated for each channel and the associated PMDF was obtained using photon replay mode [Yao et al., 2018]. The $L_{vj}$ are derived by registering anatomical (AAL) (for finger-tapping) and functional brain atlas (for working memory) to participant-specific sMRI images using SPM 12.

Table S1. Optical properties for segmented tissues.

------------------------------

Insert table S1 about here

-------------------------------

1. References:

Fang Q, Boas D (2009): Monte Carlo simulation of photon migration in 3D turbid media accelerated by graphics processing units. OPTICS EXPRESS 17:20178–20190.

Strangman G, Franceschini MA, Boas DA (2003): Factors affecting the accuracy of near-infrared spectroscopy concentration calculations for focal changes in oxygenation parameters. NeuroImage 18:865–879.

Xiao X, Yu X-T, Zhang Z, Zhao Y, Jiang Y-H, Li Z, Yang Y-H, Zhu C-Z (2018): Transcranial brain atlas. Science Advances 4:eaar6904.

Yao R, Intes X, Fang Q (2018): Direct approach to compute Jacobians for diffuse optical tomography using perturbation Monte Carlo-based photon “replay.” Biomed Opt Express 9:4588–4603.
