# Supplementary material for: Transcranial brain atlas‐based optimization for functional near‐infrared spectroscopy optode arrangement: Theory, algorithm, and application
Source: Hum Brain Mapp. 2020 Dec 17;42(6):1657–69. doi: 10.1002/hbm.25318 (PMC7978141; doi:10.1002/hbm.25318)
Supplement: Supplementary file 2 — Table S1 Optical properties for segmented tissues. [file HBM-42-1657-s002.docx]

Table S1. Optical properties for segmented tissues.

| Tissues | $\mu_{A}$ (mm^−1^) | $\mu_{S}$ (mm^−1^) | g | N |
| --- | --- | --- | --- | --- |
| Scalp | 0.0191 | 0.825 | 0.92 | 1.37 |
| Skull | 0.0136 | 1.075 | 0.92 | 1.37 |
| CSF | 0.0026 | 0.0125 | 0.92 | 1.37 |
| Gray Matter | 0.0186 | 1.3875 | 0.92 | 1.37 |
| White Matter | 0.0186 | 1.3875 | 0.92 | 1.37 |
