# Supplementary figures and images for: Transcranial brain atlas‐based optimization for functional near‐infrared spectroscopy optode arrangement: Theory, algorithm, and application
Source: Hum Brain Mapp. 2020 Dec 17;42(6):1657–69. doi: 10.1002/hbm.25318 (PMC7978141; doi:10.1002/hbm.25318)

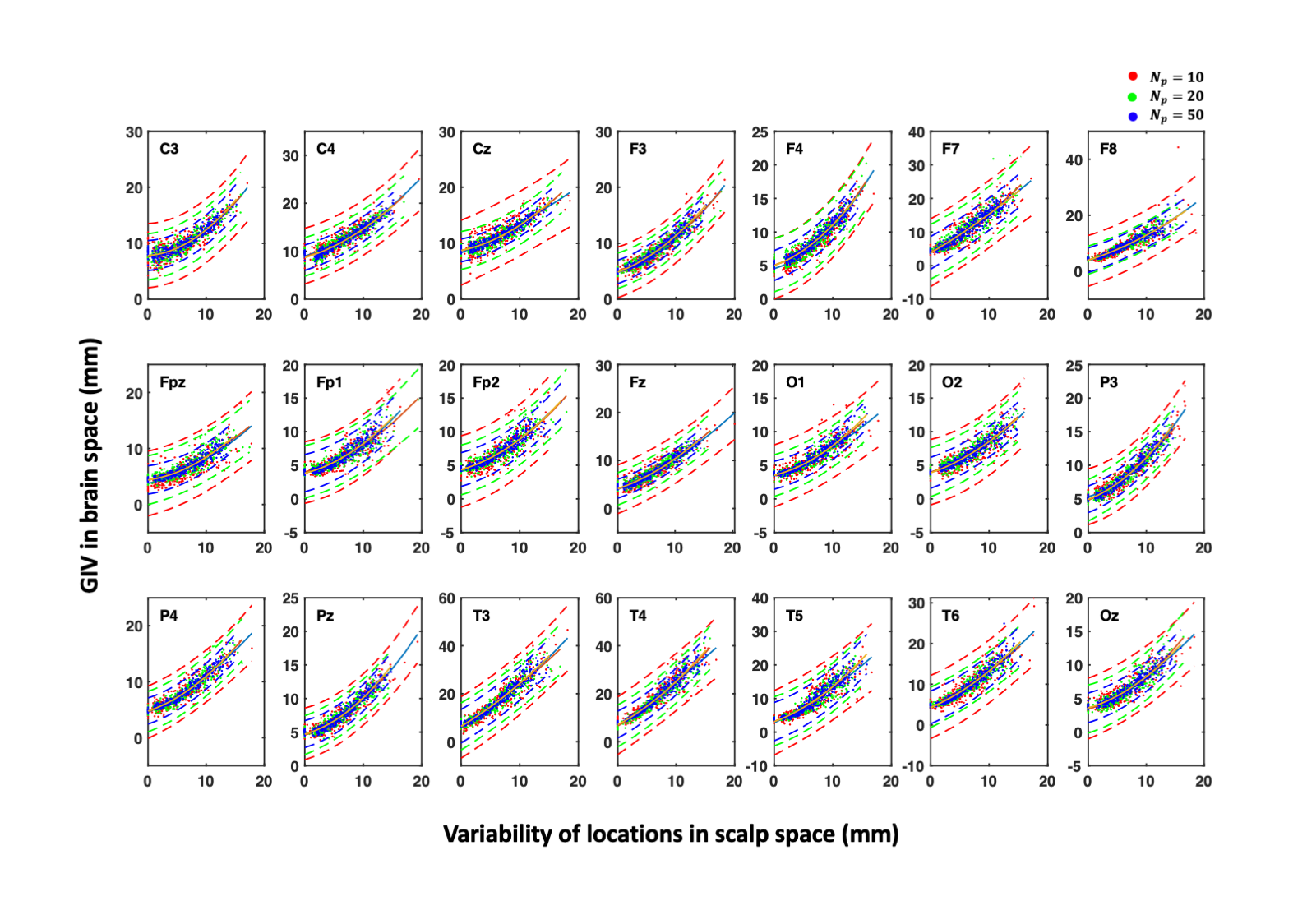

Supplement: Supplementary file 3 — Figure S1 The relationship between GIV in scalp space and that in brain space for each 10/20 scalp locations. Red, green, and blue curves represent results derived when using data from Np = 10, 20, 50 participants, respectively. The dots indicate individual values from each sampled group. The solid line and dash line represent fitted curves and prediction intervals (95%), respectively. [file HBM-42-1657-s004.tiff]

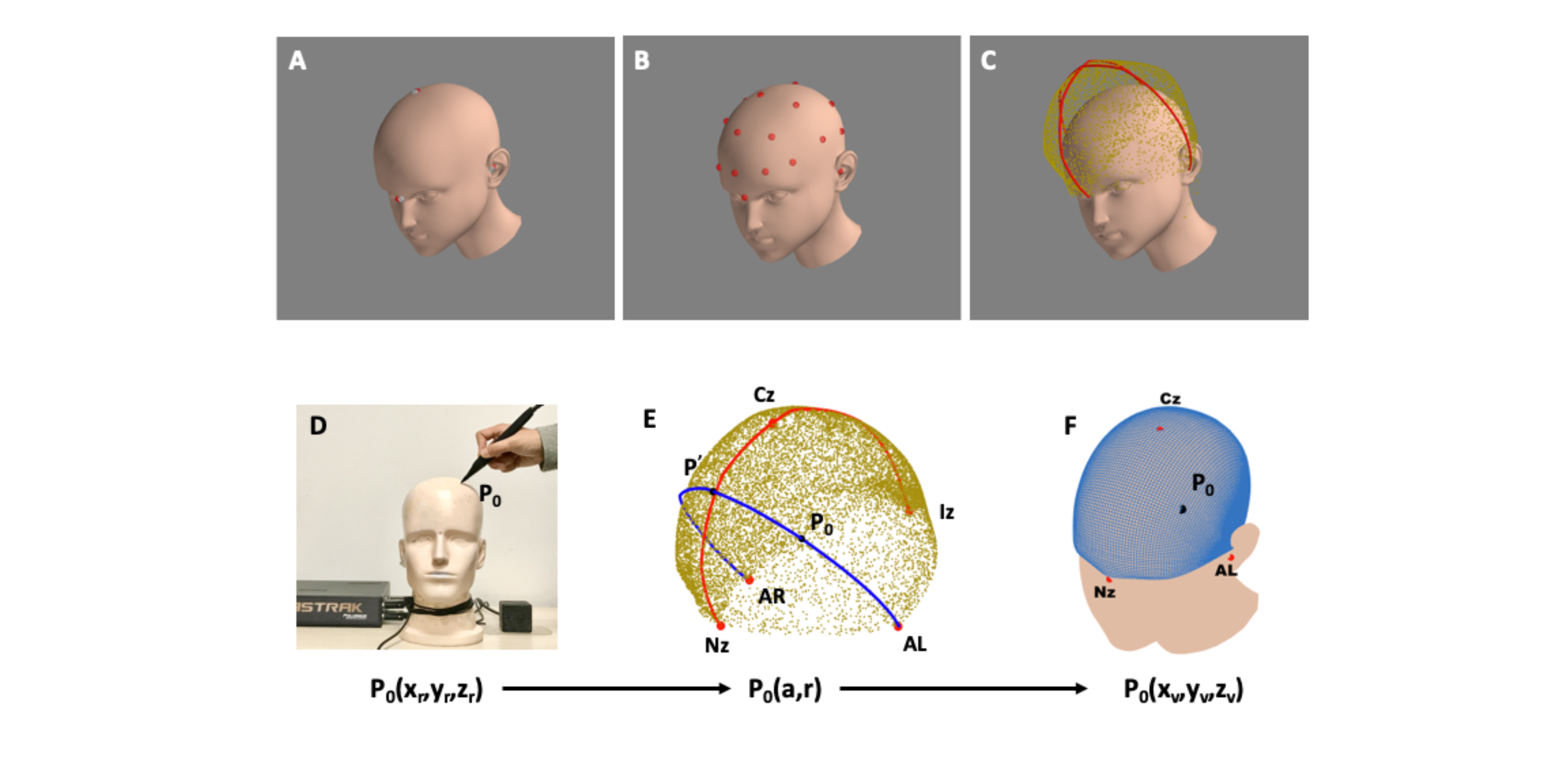

Supplement: Supplementary file 4 — Figure S2 Scalp reconstruction procedure displayed in the user interface of the navigation system (A‐C) and real‐time localization (D‐F). (A) Digitization results of 4 landmark points includes Nasion (Nz), Inion (Iz), and left/right preauricular points (AL/AR). (B) Sparse sampling of 21 points on the physical head surface. (C) Scalp reconstruction results depicted as yellow dots. The shape of reconstructed scalp is vertically stretched for better visualizing its validity. (D) An arbitrary scalp point p0 on a physical scalp model is digitized using a 3D digitizer. (E) p0 is transformed from 3D coordinates into a CPC form. (F) p0 displayed on the virtual scalp model (black dot). [file HBM-42-1657-s003.tiff]
